# Implementation of Patient Safety and Patient-Centeredness Strategies in Iranian Hospitals

**DOI:** 10.1371/journal.pone.0108831

**Published:** 2014-09-30

**Authors:** Asgar Aghaei Hashjin, Dionne S. Kringos, Jila Manoochehri, Hamid Ravaghi, Niek S. Klazinga

**Affiliations:** 1 Department of Public Health, Academic Medical Center (AMC)/University of Amsterdam, Amsterdam, the Netherlands; 2 Department of Health Services Management, School of Health Services Management and Information Sciences, Iran University of Medical Sciences, Tehran, Iran; 3 Department of Quality Improvement, Tehran Heart Center Hospital, Tehran University of Medical Sciences, Tehran, Iran; Howard University, United States of America

## Abstract

**Objective:**

To examine the extent of implementation for patient safety (PS) and patient-centeredness (PC) strategies and their association with hospital characteristics (type, ownership, teaching status, annual evaluation grade) in Iran.

**Methods:**

A cross-sectional study through an adapted version of the MARQuIS questionnaire, eliciting information from hospital and nursing managers in 84 Iranian hospitals on the implementation of PS and PC strategies in 2009–2010.

**Results:**

The majority of hospitals reported to have implemented 84% of the PS and 72% of the PC strategies. In general, implementation of PS strategies was unrelated to the type of hospital, with the exception of health promotion reports, which were more common in the Social Security Organization (SSO), and MRSA testing, which was reported more often in nonprofit hospitals. MRSA testing was also more common among teaching hospitals compared to non-teaching hospitals. The higher grade hospitals reported PS strategies significantly more frequently than lower grade hospitals. Overall, there was no significant difference in the reported implementation of PC strategies across general and specialized hospitals; except for the provision of information in different languages and recording of patient’s diet which were reported significantly more often by general than specialized hospitals. Moreover, patient hotel services were more common in private compared to public hospitals.

**Conclusions:**

Despite substantial reporting of PS and PC strategies, there is still room for strengthening standard setting on safety, patient services and patient-centered information strategies in Iranian hospitals. To assure effective implementation of PS and PC strategies, enforcing standards, creating a PS and PC culture, increasing organizational responsiveness, and partnering with patients and their families need more attention.

## Introduction

Despite the considerable developments in health care, patient safety (PS) and patient-centeredness (PC) still remain a topic of concern in health care systems world-wide [1]. Patient complaints, unsafe patient care, medical errors and adverse events are still prevalent in most health care systems, and the risk of patient harm and complications remain unacceptably high and costly in both developed and developing countries [2]–[4]. The risk of hospital related infections in some developing countries is reported to be 20 times higher than in developed countries and unsafe injections have been reported as high as 70% [5]. Up to 18% of hospitals’ inpatient admissions are associated with patient harm and 3% of them have been reported to result to death or permanent disability in some Eastern Mediterranean Region’s countries. In the United States, serious adverse events occurred in 3.7% of the hospitalizations [6]. Some countries report that patient related complications annually cost the health care budget billions [7].

Less information is available concerning PS and patient-related complaints in Iran. Over the years reports of patient harm, adverse events, medical error, unsafe injections, hazardous treatments threaten the safety of patients and result in iatrogenic complications. The Institute of Medicine estimated that up to 98,000 Americans die from medical errors annually, and hospital-associated infections cause or contribute to 99,000 deaths each year in the United States [6], [8]. An estimated 24,500 people die annually due to medical errors in Iran [9], [10]. The prevalence of hospital acquired infections is reported as high as 8–10% in Iranian hospitals [11]. A lack of attention to patients, patient involvement and the limited implementation of patient rights principles was also reported [12].

In response to the existing PS and PC problems, the Iranian Ministry of Health and Medical Education (MOHME) developed and implemented various strategies in recent years in several stages. The political agenda is currently paying more attention to the reduction of patient harm, ensuring quality, safety and the improvement of PC. To reach this mission, the MOHME statutorily implemented hospital licensing, annual hospital performance evaluations and routine inspections for all hospitals since 1997 [13]. Moreover, the MOHME compiled the Patients’ Bill of Rights in 2002 to improve patient-centered care and assed it by the Policy Council in 2009. It obliged all hospitals to implement and post the Bill in a place where it is visible to the public [12]. In addition, in 2009, the MOHME implemented “Clinical Governance” principles as a framework to improve quality of care, PS and PC in all hospitals. The MOHME also started to pilot the “Patient Safety Friendly Hospital Initiative (PSFHI)” plan for the first time in a limited number of hospitals in 2010, which was in line with WHO plans. The ambition was that these hospitals should try to obtain the first level of PSFHI standards by meeting “critical standards” [14]. Most recently, in 2011, the MOHME revised the “national hospital evaluation program” based on PS and PC principles and compiled the “Hospital Accreditation Standards in Iran” to ensure safety and improve patient-centeredness in hospitals [15]. In this manual, an extensive emphasis has been bestowed on patient safety, patient’s rights and patient-centered care in hospitals.

Although there have been some efforts to improve PS and PC in Iranian hospitals, there is very few information available on the actual implementation of specific strategies. This study therefore aims to explore:

1- The reported level of implementation of PS and PC strategies in Iranian hospitals in 2009–2010.

2- The association of the reported level of implementation of PS and PC strategies with the key characteristics of hospitals including type, ownership, teaching status and annual evaluation grade.

## Methods

This is a descriptive cross-sectional study based on a self-reported questionnaire survey. A questionnaire was distributed to hospital and nursing managers in a purposive sample of Iranian hospitals eliciting information on the implementation of PS and PC strategies in 2009 and 2010.

### The study questionnaire

Data was collected by using an existing validated (from the MARQuIS - Methods of Assessing Response to Quality Improvement Strategies – project) questionnaire [16]. We translated the questionnaire from English into Persian (Farsi). We then adapted the questions to the Iranian health care situation and added some questions on the characteristics of hospitals. We did not re-validate the questionnaire due to time and financial constraints. The questionnaire included in total 57 questions regarding the implementation of 25 PS and 32 PC strategies. The PS and PC strategies were categorized both in three groups. Each group included relevant detailed questions. Four questions on the characteristics of hospitals which are known to be influential in the implementation of PS and PC strategies were included in the questionnaire. They related to the type of hospital (multi-specialty/general, or single specialty/specialized); ownership status of hospitals (university (governmental), Social Security Organization (SSO), private for-profit and private nonprofit (including military and charity organizations); teaching status (non-teaching, non-teaching and teaching, or non-teaching, teaching and research); and the obtained annual evaluation grade (ranging from excellent, 1, 2 to 3).

### Pilot and sampling

After verifying the content of the translated and adapted questionnaire, it was piloted in 5 hospitals (including 3 public governmental, 1 private for-profit and 1 SSO hospital). Necessary changes and further improvements were made based on the responses received from the pilot hospitals. Subsequently, the questionnaires were distributed among 145 general and specialized hospitals across the country. These hospitals were selected by using a purposive sampling method and based on hospitals’ willingness to be involved in this research project.

### Statistics

We examined the extent of implementation of the selected PS and PC strategies based on the positive responses received from the respondents for specific strategies. To examine the relationship between the extent of implementation and characteristics of hospitals we conducted cross tabulations in SPSS. We applied Cramer’s V coefficient based on Pearson chi-squared test to measure association between the variables. Our criterion for the statistical differences was p<0.05.

The study was approved by the Deputy of Research and Technology of the Iran University of Medical Sciences (Code: 958/1635996).

## Results

### Study population

Of the 145 hospitals that initially participated in this study, we received questionnaires from 102 hospitals (70.3% response rate). We excluded 18 questionnaires from the final analysis due to incomplete or unreliable answers. This resulted in a total of 84 questionnaires from 72 general and 12 specialty hospitals on which we based our analysis. The characteristics of the included hospitals are shown in table 1.

**Table 1:** The characteristics of study population.

Hospitals	Ownership					Teaching status				Annual evaluation grade				
	Gov.	PFP	SSO	PNP	Total	Non-tea.	Non-tea. +Tea.	Non-tea.+ Tea.+Res.	Total	Ex.	1	2	3	Total
**General**	35	11	21	5	**72**	35	33	4	**72**	4	59	5	1	**69**
**Specialized**	11	1	0	0	**12**	1	8	3	**12**	0	12	0	0	**12**
**Total**	46	12	21	5	**84**	36	41	7	**84**	4	71	5	1	**81**

Gov. = Governmental, PFP = Private for-profit, PNP = Private nonprofit, SSO = Social Security Organization, Non-tea. = Non-teaching, Tea. = Teaching, Res. = Research, Ex. = Excellent.

The majority of hospitals were owned either by university (government) (55%) or by the SSO (25%). Forty three percent of hospitals (n = 36) were non-teaching, forty nine percent (n = 41) of them non-teaching and teaching hospitals and only seven hospitals were involved in research areas besides their non-teaching and teaching activities. Eighty five percent of hospitals were given the second highest rating in the annual evaluation program and 5% the highest rating. There is only one participating hospital that received the lowest rating (grade 3). The hospitals have on average 206 beds (range: 32–620 beds; SD = 137).

### The implementation of patient safety and patient-centeredness strategies in general

#### Patient safety strategies

From the total number of 25 PS strategies, 21 items were reported to have been highly implemented in the majority of the participating hospitals (see table 2). All hospitals acknowledged having assigned infection control personnel and to reporting hospital infections regularly. Ninety nine percent (82) of hospitals reported having a system to routinely check drug expiration dates. In contrast, a number of specific strategies for standard setting have been reported to be less implemented in hospitals. For example few hospitals reported to have specific policies to prevent patients’ falling (43%), and MRSA testing was compulsory in only 26% of hospitals. Sixteen percent of hospitals reported to have procedures in place for patient identification in the emergency department and 25% for identifying patients admitted.

**Table 2:** The extent of overall reported implementation of patient safety strategies by the type of hospitals.

Strategy		The reported implementation level of patient safety strategiesby the type of hospitals n (%)			P-value
		Total	General hospitals	Specialized hospitals	
**Assigning responsibility**	Responsible personnel for hospitalinfection control	79 (100)	67 (100)	12 (100)	–
	Responsible personnel for patient safety	51 (68.9)	42 (67.7)	9 (75)	0.559
	Responsible personnel for bloodtransfusion	64 (84.2)	56 (86.2)	8 (72.7)	0.528
	Responsible personnel for antibiotic use policy	56 (71.8)	47 (70.1)	9 (81.8)	0.723
	Responsible personnel for preventionof decubitus	50 (66.7)	40 (63.5)	10 (83.3)	0.406
	Responsible personnel for clinical waste management	68 (86.1)	58 (86.6)	10 (83.3)	0.295
	Responsible personnel forhealth promotion	68 (86.1)	56 (83.6)	12 (100)	0.318
**Specific strategies for** **standard setting**	Policies in place to prevent falls	33 (43.4)	26 (40.6)	7 (58.3)	0.523
	Hand washing policy	49 (62.8)	42 (63.6)	7 (58.3)	0.328
	MRSA testing	20 (26)	17 (26.2)	3 (25)	0.688
	Identifying patients in theemergency room	13 (15.9)	11 (15.7)	2 (16.7)	0.984
	Identifying admitted patients	21 (25)	18 (25)	3 (25)	0.701
	Availability of clinical guidelines/protocols	75 (89.3)	63 (87.5)	12 (100)	0.432
	Ratified clinical guidelines	54 (71.1)	46 (71.9)	8 (66.7)	0.096
	Updating of clinical guidelines	39 (56.5)	33 (55.9)	6 (60)	0.058
	Drug storage locked	67 (81.7)	55 (78.6)	12 (100)	0.207
	High risk drugs storage separately	71 (86.6)	62 (87.3)	9 (81.8)	0.624
	Checking drug expiration date routinely	82 (98.8)	70 (98.6)	12 (100)	0.679
**Reporting strategies** **on outcomes**	Reports on control of hospital infections	79 (100)	67 (100)	12 (100)	–
	Reports on patient safety	46 (61.3)	38 (60.3)	8 (66.7)	0.650
	Reports on blood transfusion policies	58 (75.3)	50 (75.8)	8 (72.7)	0.605
	Reports on antibiotic use policy	47 (62.7)	41 (64.1)	6 (54.5)	0.439
	Reports on prevention of decubitus	49 (64.5)	39 (60.9)	10 (83.3)	0.212
	Reports on clinical waste management	65 (85.5)	56 (86.2)	9 (81.8)	0.827
	Reports on health promotion	59 (75.6)	53 (79.1)	6 (54.5)	0.208

#### Patient-centeredness strategies

There was large variation in the reported level of implementation of various PC strategies (ranging from 13–99%). Twenty-three PC strategies (out of 32) were reported to be implemented in the majority of hospitals (see table 3).

**Table 3:** The extent of overall implementation level of patient-centeredness strategies by the type of hospitals.

Strategy		The reported implementation level of patient-centeredness strategies by the type of hospitals n (%)			P-value
		Total	Generalhospitals	Specializedhospitals	
**Patient** **services**	Possibility to contact with family or friends by patient	50 (62.5)	43 (63.2)	7 (58.3)	0.660
	Possibility to contact with family doctor or GP by patient	48 (60.8)	41 (61.2)	7 (58.3)	0.618
	Providing meals for family and relatives of patients	67 (81.7)	57 (81.4)	10 (83.3)	0.875
	Providing room/bed for family and relatives of patients	56 (67.5)	50 (70.4)	6 (50)	0.245
	Offering single room upon request	40 (48.8)	36 (51.4)	4 (33.3)	0.269
	Access to internet in or outside the room	15 (18.1)	13 (18.3)	2 (16.2)	0.907
	Access to tel. in the room with instructions in other languages	54 (65.1)	45 (63.4)	9 (75)	0.672
	Access to TV and satellite in the room	68 (82.9)	56 (80)	12 (100)	0.235
	Providing daily newspaper for patients	12 (14.5)	9 (12.7)	3 (25)	0.434
	Access to smoking room	11 (13.4)	9 (12.9)	2 (16.7)	0.865
	Having coordinator for patients affairs	46 (55.4)	40 (56.3)	6 (50)	0.650
	Having coordinator for discharge of patients	44 (53)	39 (54.9)	5 (41.7)	0.389
	Having transport services for patients	17 (20.5)	14 (19.7)	3 (25)	0.894
	Providing medicines if needed after discharge	73 (88)	62 (87.3)	11 (91.7)	0.355
	Recording of patient’s diet preferences	82 (97.6)	71 (98.6)	11 (91.7)	0.045
	Offering a choice of the meals to patients	19 (22.6)	16 (22.2)	3 (25)	0.764
	Offering a choice in the timing of the meals	13 (15.5)	12 (16.7)	1 (8.3)	0.502
	Visit of patients by family or relatives	80 (96.4)	69 (95.8)	11 (100)	0.788
**Patient information,** **involvement and** **empowerment**	Providing information in different languages	67 (79.8)	60 (83.3)	7 (58.3)	0.016
	Written policy for patient involvement in decision making	58 (69.9)	51 (71.8)	7 (58.3)	0.625
	Possibility to give information to patients in their language	67 (79.7)	57 (79.1)	10 (83.3)	0.470
	Having a procedure for the requirements before admission	40 (48.8)	35 (49.3)	5 (41.7)	0.408
	Possibility to contact with the patient’s doctor before admission	12 (14.3)	9 (12.5)	3 (25)	0.227
	Patients and their family involvement in care decision making	51 (60.7)	43 (59.7)	8 (66.7)	0.856
	Providing written information regarding to patient’s treatment	43 (51.2)	35 (48.6)	8 (66.7)	0.264
	Written policy for informed consent to interventions/treatments	74 (89.2)	63 (88.7)	11 (91.7)	0.763
**Patient** **rights**	Have patient rights department	61 (73.5)	51 (71.8)	10 (83.3)	0.631
	Written policy regarding confidentiality of patient information	78 (92.9)	66 (91.7)	12 (100)	0.584
	Written policy for patients’ privacy	79 (95.2)	67 (94.4)	12 (100)	0.701
	Written policy for patients’ access to their health record	80 (96.4)	68 (95.8)	12 (100)	0.468
	Written policy for appropriate religious support	75 (90.4)	64 (90.1)	11 (91.7)	0.425
	Patient rights posted in a place visible to all patients and visitors	83 (98.8)	71 (98.6)	12 (100)	0.681

The strategies related to patient rights had the highest reported implementation rate (>89%). The least implemented strategy in this group was having a separate patient rights department, which was present in74% of the hospitals. The provision of some patient and family hotel services (including access to internet, daily newspaper, smoking room, transport services, and choice and timing of the meals) were rarely implemented in hospitals (<23% reported implementation rate). In contrast, some other hotel services such as access to telephone and TV in the room were more common among (65% and 83% respectively) the hospitals.

### The association between implementation of patient safety or patient-centeredness strategies and characteristics of hospitals

#### Patient safety strategies and characteristics of hospitals

The implementation of PS strategies appears unrelated to the type of hospital (table 4). However, the SSO hospitals reported implementation of reports on health promotion significantly more often than the total average reported rates. The hospitals owned by nonprofit organizations reported MRSA testing significantly more often than the total average rate of all hospitals (table 5). The hospitals that are not involved in teaching and research activities, reported the presence of the antibiotic use policy significantly more often than the hospitals involved in teaching and research activities. In contrast, table 6 shows MRSA testing is reported significantly more often by hospitals involved in research, besides their teaching and therapeutic activities. The differences in the implementation rates of the majority of PS strategies were not associated with differences in hospital grades. However, the hospitals with a higher grade reported significantly more often to having responsible personnel available for clinical waste management and health promotion. Higher grade hospitals reported more often to have clinical waste management procedures in place and to perform health promotion activities than lower grade hospitals (see table 7).

**Table 4:** The associations between implementation of patient safety and patient-centeredness strategies and the type of hospitals.

Strategy		Extent of reported implementation level of patient safety and patient-centeredness strategies by the type of hospitals n (%)			P-value (Cramer’sV coefficient)
		Total	General hospitals	Specialized hospitals	
**Patient** **safety**	–	–	–	–	–
**Patient-** **centeredness**	Providing information indifferent languages	67 (79.8)	60 (83.3)	7 (58.3)	0.016 (0.31)
	Recording of patient’sdiet preferences	82 (97.6)	71 (98.6)	11 (91.7)	0.045 (0.27)

**Table 5:** The associations between implementation of patient safety and patient-centeredness strategies and the ownership of hospitals.

Strategy		Extent of reported implementation level of patient safety and patient-centerednessstrategies by the ownership of hospitals n (%)					P-value (Cramer’sV coefficient)
		Total	University	SSO	PFP	PNP	
**Patient safety**	Reports on health promotion	59 (75.6)	26 (65)	20 (95.2)	11 (91.7)	(40)	0.019 (0.31)
	MRSA testing	20 (26)	10 (22.2)	2 (11.1)	4 (44.4)	4 (80)	0.033 (0.30)
**Patient-** **centeredness**	Providing room/bed for family and relativesof patients	56 (67.5)	23 (50)	16 (80)	12 (100)	5 (100)	0.012 (0.31)
	Access to internet	15 (18.1)	8 (17.4)	1 (5)	3 (25)	3 (60)	0.021 (0.30)
	Having coordinator for patients affairs	46 (55.4)	22 (47.8)	13 (65)	9 (75)	2 (40)	0.020 (0.30)
	Possibility to contact with the patient’s doctorbefore admission	12 (14.3)	5 (10.9)	0 (0)	5 (41.7)	2 (40)	0.025 (0.29)
	Offering a choice of the meals to patients	19 (22.6)	6 (13)	0 (0)	10 (83.3)	3 (60)	0.000 (0.50)
	Offering a choice in the timing of the meals	13 (15.5)	6 (13)	1 (4.8)	5 (41.7)	1 (20)	0.006 (0.33)

PFP = private for-profit; PNP = private nonprofit.

**Table 6:** The associations between implementation of patient safety and patient-centeredness strategies and the teaching and research status of hospitals.

Strategy		Extent of reported implementation level of patient safety and patient-centeredness strategies by the teaching status of hospitals n (%)				P-value (Cramer’sV coefficient)
		Total	Non-teaching	Non-teaching& teaching	Non-teaching &teaching & research	
**Patient** **safety**	Reports on antibioticuse policy	47 (62.7)	23 (76.7)	21 (53.8)	3 (50)	0.020 (0.28)
	MRSA testing	20 (26)	6 (19.4)	10 (25.6)	4 (57.1)	0.001 (0.35)
**Patient-** **centeredness**	Providing dailynewspaperfor patients	12 (14.5)	4 (11.4)	4 (9.8)	4 (57.1)	0.021 (0.26)
	Having coordinatorfor patientsaffairs	46 (55.4)	26 (74.3)	18 (43.9)	2 (28.6)	0.002 (0.32)
	Having transportservices forpatients	17 (20.5)	7 (20)	6 (14.6)	4 (57.1)	0.007 (0.29)

**Table 7:** The associations between implementation of patient safety and patient-centeredness strategies and the annual evaluation grade of hospitals.

Strategy		Extent of reported implementation level of patient safety and patient-centeredness strategies by the annual evaluation grade of hospitals n (%)					P-value (Cramer’s V coefficient)
		Total	Excellent1	Grade 1	Grade 2	Grade 3	
**Patient safety**	Responsible personnel for clinical waste management	65 (85.5)	4 (100)	58 (86.6)	3 (75)	0 (0)	0.022 (0.31)
	Responsible personnel for health promotion	65 (85.5)	4 (100)	58 (86.6)	3 (75)	0 (0)	0.003 (0.36)
	Reports on clinical waste management	62 (84.9)	4 (100)	55 (85.9)	3 (75)	0 (0)	0.001 (0.39)
	Reports on health promotion	56 (74.7)	4 (100)	49 (74.2)	3 (75)	0 (0)	0.04 (0.30)
	Availability of clinical guidelines/protocols	73 (90.1)	2 (50)	66 (93)	4 (80)	1 (100)	0.042 (0.28)
**Patient-centeredness**	–	–	–	–	–	–	–

#### Patient-centeredness strategies and characteristics of hospitals

There is no significant difference between general and specialized hospitals in the reported implementation rates of PC strategies with the exception of providing information in different languages and recording of the patient’s diet preference. The general hospitals reported significantly more often to have implemented these strategies than specialized hospitals (see table 4). The patient services strategies including provision of a room/bed for the relatives of patients, possibility to contact the patient’s family doctor/specialist before admission, offering a choice of meals and timing of meals to patients, were reported to be implemented significantly more often in private (both for-profit and nonprofit) hospitals compared to the total average rate of all hospitals (as shown in the table 5). The association was strongest (Cramer’s V = 0.50) in case of offering a choice of the meals to patients in the private hospitals (83% compared to average rate of 23%). It seems that the rate of implementation of PC strategies is unrelated to the annual evaluation grade of hospitals.

## Discussion

This is the first study to our knowledge that provides comprehensive insight in the (reported) implementation of PS and PC strategies in Iranian hospitals. However, the study has some limitations. First, the authors tried to include a representative sample of hospitals in the study as much as possible. Although the sample size of the study was relatively small especially with regard to the limited number of hospitals with lower annual evaluation grades (grade 2 and 3) and the hospitals owned by nonprofit organizations, the total number of these groups of hospitals is limited in the country. Another limitation concerns the validity of the questionnaire. Due to time and funding constraints, the questionnaire was not re-validated, which seems acceptable given the minor changes that were made compared to the original validated version. Another limitation of the study was the 30% non-response and those who had to be excluded from the final analysis due to incomplete or unreliable data. Finally, the study was based on a purposive sampling method and a self-reported questionnaire; potentially producing biased results.

Our study identified that the strategies related to assigning responsibilities, outcomes reporting, patient rights and the majority of patient services were reported to be most often implemented by all hospitals. However, the specific strategies which were related to standard setting and some patient hotel services were reported less commonly in Iranian hospitals. These findings are mostly in line with the MARQuIS study [17], [18] which reported similar results concerning the implementation of PS and PC strategies in hospitals in European countries (Belgium, the Czech Republic, France, Ireland, the Netherlands, Poland, Spain, UK). Surprisingly, this study also reported that MRSA testing and choice of timing of the meals were less common strategies in European countries. Our findings related to patient identification strategies are different from the MARQuIS study. We found patient identification strategies were less commonly applied in Iranian hospitals, while in the MARQuIS study a relatively higher implementation rate was reported. Our study has also found a meaningful association between ownership status and annual evaluation grade of hospitals and the implementation of PS and PC strategies. The strategies which are (statutorily) part of the Iranian annual evaluation program were more often reported to be implemented compared to the other non-obligatory strategies. In addition, the rate of implementation for (some of) the patient hotel services was significantly more often reported in the private (both for-profit and nonprofit) hospitals compared to other hospitals.

### Attention to the specific strategies for standard setting and patient hotel services

Although the majority of PS and PC strategies assessed in our study were reported to be widely implemented, there were less frequent implementation rates reported for strategies related to standard setting and (some) patient services. Identifying patients in hospital, MRSA testing, policies for preventing patient falls were less common PS strategies reported to be implemented by hospitals. Moreover, the implementation of a number of patient hotel services was reported to be significantly lower in hospitals compared to other strategies. Although our findings are in line with the lower implementation rates, some PS and PC strategies reported in a few countries [17]–[19]. There is a concern about suboptimal implementation rates. More attention needs to be given to the implementation of these strategies to complete the cycle of PS and PC care in hospitals. These strategies have been reported in other studies to be important factors in improving safety and PC in hospitals. They have also been identified as the main PS and PC issues in hospitals around the world [20]–[24].

### Emphasize the effectiveness of strategies in practice

Although the overall reported implementation rate of the majority of the PS and PC strategies was relatively high, a continuous debate concerning the actual impact of these strategies for improving safety and PC in hospitals. A gap remains between the reported implementation rate and the effectiveness of strategies in practice. The evidence from the relevant literature shows that the strategies in some cases have not been effectively implemented. For instance, although 100% of hospitals in our study reported having responsible personnel and routinely reports for infection control in place, several studies identified that the hospital infection rate in Iran is still remarkably higher than the infection rate in European countries (8–10% compared to 5%) [25], [26]. In addition, almost all hospitals reported to having posted the Patients’ Bill of Rights and to implementing the patient rights principles. Some report that patient rights principles are not fully implemented by all health care providers [27]. Our study thus suggests that reporting a higher rate of strategies implementation does not guarantee safety and better patient care. Such efforts should be supported by legal embedding and enforcement of strategies, creating an organizational responsiveness and culture of safety and PC [28]–[30]. Truly partnering with patients and their families can also be effective [2], [30], [31], [32].

### How is the implementation of patient safety and patient-centeredness strategies associated with characteristics of hospitals?

Our study showed that there are meaningful associations between the implementation of PS and PC strategies and the characteristics of hospitals, which is in line with related research in this field. A study has revealed that the hospital characteristics may predict the implementation of PS and PC strategies [33]. In our study the implementation rates of patient hotel services were reported significantly more often in the private (for-profit and nonprofit) hospitals. This may suggest that private hospitals in Iran are more service-oriented and thus more interested in implementation of hotel services. The financial incentives for hospitals clearly play a role in the implementation of PC strategies especially with regard to hotel services. The results showed that the hospitals were involved in teaching and research activities besides non-teaching activities, reported higher implementation of some specific PS strategies (i.e. MRSA testing). Although it is difficult to speak of a pattern because of the limited number of the hospitals involved in both non-teaching, teaching and research activities, these hospitals appear to be more safety-oriented.

Finally, our results revealed that the hospitals with higher evaluation grades, reported a significantly higher implementation rate of the strategies compared to the lower grade hospitals. Hospitals in our study reported a higher implementation rate with regard to obligatory PS strategies which were subjected to the Iranian hospital evaluation program. The commitment to PS strategies by the higher graded hospitals is required in order to achieve a higher evaluation grade. The obligation of hospitals to implement specific strategies can be a potential incentive for planning and implementing PS or PC strategies.

## Conclusion

Although the implementation of a number of PS and PC strategies were widely reported by Iranian hospitals, there is room for improvement and strengthening of the implementation of specific strategies related to standard setting and patient services. The association of PS and PC strategies with characteristics of hospitals (type, ownership, teaching status and annual evaluation grade) provides a mixed picture. The implementation of PS and PC strategies are influenced by the characteristics of these hospitals. The safety strategies which are statutorily obligated by the government were more frequently implemented in the higher grade hospitals. The PC strategies were more common in the private (for-profit and nonprofit) hospitals, which appear to be more service-oriented. Despite the reporting of relatively high implementation rates for the majority of strategies, the effectiveness of PS and PC strategies in hospitals still needs improvement. An effective implementation of PS and PC initiatives may depend on the legal embedding and enforcement of standards, creating an organizational responsiveness to demands of patients, creating a PS and PC culture in hospitals and partnering with patients and their families.

## References

[1] World Health Organization (WHO) (2013) Patient safety assessment manual. Available: http://applications.emro.who.int/dsaf/emropub_2011_1243.pdf. Accessed 9 August 2013.

[2] Australian Commission on Safety and Quality in Health Care (ACSQHC) (2010) Patient-centred care: improving quality and safety by focusing care on patients and consumers, Discussion paper draft for public consultation, September. Available: http://www.podiatrywa.com.au/news/146-Patient-CentredCarePaperweb.pdf. Accessed 13 February 2013.

[3] Institute for Patient- and Family-Centered Care website (2013) Available: http://www.ipfcc.org. Accessed 7 July 2013.

[4] Wong J, Beglaryan H (2004) Strategies for Hospitals to Improve Patient Safety. A Review of the Research February, The Change Foundation. Available: http://www.providence.on.ca/wp-content/uploads/2012/05/Change-Foundation-Improve-Patient-Safety.pdf. Accessed 14 February 2013.

[5] World Health Organization (WHO) (2013) 10 facts on Patient Safety. Available: http://www.who.int/features/factfi les/patient_safety/en/index.html. Accessed 8 June 2013.

[6] James JT (2013) A New, Evidence-based Estimate of Patient Harms Associated with Hospital Care, Journal of Patient Safety: September; Volume 9 - Issue 3 - p 122–128 doi: 10.1097/PTS.0b013e3182948a69. 10.1097/PTS.0b013e3182948a69 23860193

[7] World Health Organization (WHO): Patient safety (2013) Available: http://www.emro.who.int/entity/patient-safety/. Accessed 7 June 2013.

[8] Pollack A (2010) Rising Threat of Infections Unfazed by Antibiotics. New York Times, Feb. 27.

[9] Zargarzadeh AH (2013) Medication Safety in Iran. J Pharm Care 1(4): 125–126.

[10] Sheikhtaheri A, Sadoughi F, Ahmadi M, Moghaddasi H (2013) Developing Iranian patient safety indicators: an essential approach for improving safety of health care. Asian Biomedicine June; Vol. 7 No. 3, 365–373, doi: 10.5372/1905-7415.0703.188.

[11] Zahraei SM, Eshrati B, Masoumi Asl H, Pezeshki Z (2012) Epidemiology of Four Main Nosocomial Infections in Iran during March 2007– March 2008 based on the Findings of a Routine Surveillance System. Arch Iran Med 15(12): 764–766. 23199249

[12] Joolaee S, Hajibabaee F (2012) Patient rights in Iran: A review article. Nurs Ethics 19: 45 originally published online 2 December 2011, doi:10.1177/0969733011412100. 10.1177/0969733011412100 22140178

[13] Aghaei Hashjin A, Delgoshaei B, Kringos DS, Tabibi SJ, Manoochehri J, et al.. (2014) Implementation of hospital quality assurance policies in Iran. Submitted for publication in 2014. 10.1108/IJHCQA-03-2014-0034 25982635

[14] World Health Organization (WHO): Patient safety friendly hospitals (2013) Available: http://www.emro.who.int/patient-safety/countries/country-activities-islamic-republic-of-iran.html. Accessed at 10 June 2013.

[15] Iranian Ministry of Health and Medical Education (MOHME) (2011) Hospital Accreditation Standards in Iran. Tehran.

[16] Assessing of the effectiveness of quality improvement strategies in Europe: the MARQuIS project (2006) Available: http://www.marquisquestionnaire.nl/marquis/print/printallgeneral.asp. Accessed 25 July 2006.

[17] Lombarts MJ, Rupp I, Vallejo P, Suñol R, Klazinga NS (2009) Application of quality improvement strategies in 389 European hospitals: results of the MARQuIS project. BMJ journal of Quality & Safety in Health Care, vol. 18, supplement 1. 10.1136/qshc.2008.029363 PMC3269892 19188458

[18] Groene O, Lombarts MJ, Klazinga N, Alonso J, Thompson A, Suñol R (2009) Is patient-centredness in European hospitals related to existing quality improvement strategies? Analysis of a cross-sectional survey (MARQuIS study). BMJ journal of Quality & Safety in Health Care, vol. 18 supplement 1: i44–i50 doi:10.1136 10.1136/qshc.2008.029397 PMC2629879 19188461

[19] Lombarts MJ, Rupp I, Vallejo P, Klazinga NS, Sunol R (2009) Differentiating between hospitals according to the “maturity” of quality improvement systems: a new classification scheme in a sample of European hospitals. BMJ journal of Quality & Safety in Health Care, vol. 18, supplement 1. 10.1136/qshc.2008.029389 PMC2629850 19188460

[20] Griese BR, Frazier CR (2011) Patient Identification: Simple Measures, Big Benefits: Ensuring patient identification means avoiding financial loss and accreditation - and keeping patients safe. Advance health care network, February 17. Available: http://healthcare-executive-insight.advanceweb.com/Columns/Case-Files-Legal-Issues-for-Healthcare-Executives/Patient-Identification-Simple-Measures-Big-Benefits.aspx. Accessed 23 September 2013.

[21] World Health Organization (WHO) (2007) Patient identification. Patient Safety Solutions, vol. 1, solution 2, May.

[22] Deleo FR, Otto M, Kreiswirth BN, Chambers HF (2010) Community-associated meticillin-resistant Staphylococcus aureus. Lancet 375: 1557–1568. 20206987 10.1016/S0140-6736(09)61999-1 PMC3511788

[23] Askari E, Soleymani F, Arianpoor A, Tabatabai SM, Amini AR, et al (2012) Epidemiology of mecA-Methicillin Resistant Staphylococcus aureus (MRSA) in Iran: A Systematic Review and Meta-analysis. Iranian Journal of Basic Medical Sciences Sep-Oct 15(5): 1010–1019. PMC3586924 23493646

[24] Grundmann H, Aires-de-Sousa M, Boyce J, Tiemersma E (2006) Emergence and resurgence of methicillinresistant Staphylococcus aureus as a public-health threat. Lancet 368: 874–85. 16950365 10.1016/S0140-6736(06)68853-3

[25] Zahraei SM, Eshrati B, Masoumi Asl H, Pezeshki Z (2012) Epidemiology of Four Main Nosocomial Infections in Iran during March 2007– March 2008 based on the Findings of a Routine Surveillance System. Arch Iran Med 15(12): 764–766. 23199249

[26] Askarian M, Gooran NR (2003) National nosocomial infection surveillance system-based study in Iran: additional hospital stay attributable to nosocomial infections. American Journal of Infection Control, Dec 31(8): 465–8. 10.1016/s0196-6553(03)00673-4 14647108

[27] Joolaee S, Hajibabaee F (2011) Patient rights in Iran: A review article. Nurs Ethics 2012 19: 45 10.1177/0969733011412100 22140178

[28] Wagner C, Smits M, Sorra J, Huang CC (2013) Assessing patient safety culture in hospitals across countries. International Journal of Quality in Health Care Jul 25(3): 213–21 doi: 10.1093 10.1093/intqhc/mzt024 PMC3671738 23571748

[29] Aghaei Hashjin A, Ravaghi H, Kringos DS, Ogbu UC, Fischer C, et al.. (2014) Using Quality Measures for Quality Improvement: The Perspective of Hospital Staff. PLOS ONE, January 23, DOI: 10.1371/journal.pone.0086014. 10.1371/journal.pone.0086014 PMC3900447 24465842

[30] Verbeek-Van Noord I, Wagner C, Twisk JWR, De Bruijne MC (2013) Is culture associated with patient safety in the emergency department? A study of staff perspectives, Int J Qual Health Care (2014) 26 (1): 64–70 first published online December 10, doi:10.1093/intqhc/mzt087. 10.1093/intqhc/mzt087 24334232

[31] Groene O, Sunol R, Klazinga NS, Wang A, Dersarkissian M, et al.. (2014) Involvement of patients or their representatives in quality management functions in EU hospitals: implementation and impact on patient-centred care strategies, Int J Qual Health Care first published online March 9, doi:10.1093/intqhc/mzu022. 10.1093/intqhc/mzu022 PMC4001693 24615596

[32] Hrisos S, Thomson R (2013) Seeing It from Both Sides: Do Approaches to Involving Patients in Improving Their Safety Risk Damaging the Trust between Patients and Healthcare Professionals? An Interview Study. PLOS ONE, November 06, DOI: 10.1371/journal.pone.0080759. 10.1371/journal.pone.0080759 PMC3819291 24223230

[33] Longo DR, Hewett JE, Ge B, Schubert S (2007) Hospital patient safety: characteristics of best-performing hospitals. Journal of Healthcare Management May-Jun 52(3): 188–204 discussion 204–5. 17552355

